# Solid-Supported Proteins in the Liquid Chromatography Domain to Probe Ligand-Target Interactions

**DOI:** 10.3389/fchem.2019.00752

**Published:** 2019-11-15

**Authors:** Marcela Cristina de Moraes, Carmen Lucia Cardoso, Quezia Bezerra Cass

**Affiliations:** ^1^Laboratório SINCROMA, Instituto de Química, Departamento de Química Orgânica, Universidade Federal Fluminense, Niterói, Brazil; ^2^Grupo de Cromatografia de Bioafinidade e Produtos Naturais, Departamento de Química, Faculdade de Filosofia, Ciências e Letras de Ribeirão Preto, Universidade de São Paulo, Ribeirão Preto, Brazil; ^3^Separare, Departamento de Química, Universidade Federal de São Carlos, São Carlos, Brazil

**Keywords:** bioaffinity chromatography, ligand screening, ligand-target interactions, zonal bioaffinity chromatography, frontal bioaffinity chromatography, ligand fishing

## Abstract

Ligand-target interactions play a central role in drug discovery processes because these interactions are crucial in biological systems. Small molecules-proteins interactions can regulate and modulate protein function and activity through conformational changes. Therefore, bioanalytical tools to screen new ligands have focused mainly on probing ligand-target interactions. These interactions have been evaluated by using solid-supported proteins, which provide advantages like increased protein stability and easier protein extraction from the reaction medium, which enables protein reuse. In some specific approaches, precisely in the ligand fishing assay, the bioanalytical method allows the ligands to be directly isolated from complex mixtures, including combinatorial libraries and natural products extracts without prior purification or fractionation steps. Most of these screening assays are based on liquid chromatography separation, and the binding events can be monitored through on-line or off-line methods. In the on-line approaches, solid supports containing the immobilized biological target are used as chromatographic columns most of the time. Several terms have been used to refer to such approaches, such as weak affinity chromatography, high-performance affinity chromatography, on-flow activity assays, and high-performance liquid affinity chromatography. On the other hand, in the off-line approaches, the binding event occurs outside the liquid chromatography system and may encompass affinity and activity-based assays in which the biological target is immobilized on magnetic particles or monolithic silica, among others. After the incubation step, the supernatant or the eluate from the binding assay is analyzed by liquid chromatography coupled to various detectors. Regardless of the selected bioanalytical approach, the use of solid supported proteins has significantly contributed to the development of automated and reliable screening methods that enable ligands to be isolated and characterized in complex matrixes without purification, thereby reducing costs and avoiding time-laborious steps. This review provides a critical overview of recently developed assays.

## Introduction

The use of solid-supported proteins to evaluate protein-protein interactions and to purify proteins is a well-founded tool (Muronetz et al., 2001; Perret and Boschetti, 2018). Interactions between proteins and small molecules have also been well-explored (de Moraes et al., 2014a; Zheng et al., 2014; Hage, 2017). More recently, solid-supported proteins have been employed not only to assess these interactions, but mainly as a strategy to isolate small molecules from complex combinatory libraries (Forsberg and Brennan, 2014; Zhuo et al., 2016; Vanzolini et al., 2018a; Wang L. et al., 2018). These applications are based on the principle of specific and reversible affinity interactions with the immobilized target protein and correlate well with zonal and frontal chromatography. For this reason, various chromatographic terms have been used.

Terms such as weak affinity chromatography (WAC) (Meiby et al., 2013; Singh P. et al., 2017; Ohlson and Duong-Thi, 2018; Lecas et al., 2019), high-performance affinity chromatography (HPAC) (Hage, 2017; Li Z. et al., 2017; Beeram et al., 2018; Zhang C. et al., 2018), high-performance liquid affinity chromatography (HPLAC) (Zheng et al., 2014), biointeraction chromatography (Wainer, 2004), affinity monolith chromatography (AMC) (Lecas et al., 2019), and cellular chromatography (CC) (Ciesla et al., 2016; Xu et al., 2019) have been largely and correctly employed, but all of them could be well-settled in the general term bioaffinity chromatography, which has been introduced to differentiate them from the classic affinity chromatography (de Moraes et al., 2016).

Moreover, the diverse ways to use solid-supported proteins have created many protocol possibilities and in some-cases it is misleading to name it as chromatography. The problem clearly is not the adopted name, but confusion arises when one misses a reference for not using the correct acronyms. Another problem is when the procedure is named chromatography just because it uses solid-supported proteins, as in the case of some off-line devices, like bio-SPE (Forsberg and Brennan, 2014; Forsberg et al., 2014), fishing (Wang L. et al., 2018; Xu et al., 2019; Zhang et al., 2019), and “functional chromatography” (Kang et al., 2014; Lau et al., 2015). Off-line assays usually aim to identify protein ligands from a mixture with chemical characterization of the isolated (fished) ligands.

Zonal bioaffinity chromatography has been used in several assays to measure the affinity of a given ligand toward a certain protein, as depicted in numerous comprehensive reviews (Jonker et al., 2011; Hage, 2017; Tao et al., 2018b). One of the nicest applications comes from chiral protein columns for assessing interactions of a given enantiomer with its binding site, as with (S)-lorazepam hemisuccinate separation, which was dramatically affected by the use of (S)-warfarin in the mobile phase. This result was used to demonstrate the allosteric interaction between the binding sites of these chiral drugs. The presence of (S)-warfarin in the mobile phase affected the chiral separation of the lorazepam hemisuccinate racemate (Domenici et al., 1991). Competitive binding experiments can be employed to evaluate whether allosteric interactions are cooperative or anti-cooperative (Wainer, 2004). Furthermore, the use of human serum albumin (HSA) column allows the equilibrium between free and bound solutes to be directly determined and can help to monitor how interaction between different ligands changes the protein binding properties. Zonal elution provides information about ligand binding sites in the protein, and the obtained data might also be applied in studies about structure–retention relationships (QSRRs) (Bertucci et al., 2003; Bertucci and Domenici, 2012). Zonal bioaffinity chromatography has created countless possibilities to study protein-solute interactions by means of linear or non-linear elution data (Jozwiak et al., 2002; Vanzolini et al., 2013b; Zheng et al., 2014; Tao et al., 2018b).

In the realm of immobilized enzymes, kinetic parameters can be measured by on-line assays (De Simone et al., 2019); for example, assays with glyceraldehyde-3-phosphate dehydrogenase (GAPDH) (Cardoso et al., 2006) and purine nucleoside phosphorylase (PNP) (de Moraes et al., 2013). These assays allow not only the activity parameters of the immobilized enzyme to be gathered, but also to screen inhibitors (Vanzolini et al., 2013a; Rodrigues et al., 2016) or substrates (Calleri et al., 2014) and to unveil inhibition mechanisms, even for tight inhibitors (Rodrigues et al., 2015).

Frontal bioaffinity chromatography has been employed in thermodynamic and kinetic analyses and can simultaneously provide information about the amount of immobilized protein, the number of active binding sites, and the equilibrium constants of these sites (Lecas et al., 2019). Additionally, the molecular interaction can be identified and characterized in a concentration-independent manner, and, in a mixture, it can be revealed in the range from millimolar to picomolar dissociation constants (Schriemer, 2008; Calleri et al., 2009; Temporini et al., 2013; Hage, 2017). These are probably the main benefits of frontal elution when it comes to assessing molecular interactions in complex combinatory library as compared to zonal elution (Michel et al., 2013).

**Figure 1:**
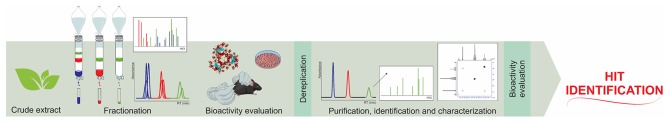
Classic bio-guided assay work chart for hit identification in a combinatorial natural product library.

The classic approaches to isolating ligand hits selectively and identifying them in natural product libraries are not easy and are usually troublesome (Figure 1).

The diverse molecular frameworks that are present in a natural product extract are the major bottleneck regarding ligand identification in this matrix. Therefore, many affinity-based screening platforms have been proposed to overcome the tiresome classic approach. In this context, screening assays carried out in a static fashion (Vanzolini et al., 2018b) have been considered as the most interesting means of identifying or isolating ligand hits from complex natural libraries (Cieśla and Moaddel, 2016; Zhuo et al., 2016; Fu et al., 2019).

A critical overview of the use of solid-supported proteins in the domain of chromatography, to disclose interactions between proteins and small molecules, will be discussed herein. In this respect, we will cover zonal and frontal bioaffinity chromatography as the on-line methods, and we will also discuss the off-line approaches. This review covers articles published after 2014 (de Moraes et al., 2014b).

## On-Line Approaches

Various solid supports and different immobilization protocols can be used to create bioaffinity stationary phases. Suitable supports include open tubular and packed fused silica capillaries (da Silva et al., 2012; de Moraes et al., 2014a; Wang L. et al., 2018), monolithic supports (Pfaunmiller et al., 2013; Kubota et al., 2017), immobilized artificial membranes (IAM) (Habicht et al., 2015; Yang Y.-X. et al., 2017), silica (Liu G. et al., 2017; Li Z. et al., 2017), and other polymeric and particulate supports. Specificity is the most important characteristic of a solid support: to avoid false positives, the support should not interact with the sample components. Secondary interactions can be examined with a control column, which contains the inactive/denatured target protein or only the solid support that is used to immobilize the target protein. Employing non-ligands as negative controls is another strategy to assess secondary interactions.

In the case of on-line approaches, where the binding event occurs within the liquid chromatography system (i.e., in a chromatographic column), the material that is used as solid support should be mechanically and chemically stable, have rapid mass transfer capacity, and display low backpressure and adequate efficiency. In addition, the solid support should be able to retain the target protein even in the on-flow conditions, while the immobilized target protein should retain the ligand and separate it from the other sample components.

Solid supports can be derivatized with several functional groups, so that countless immobilization procedures can be used to prepare the protein (biological target)-containing stationary phase. One of the most frequently employed methods is to attach primary amines, epoxides, aldehydes, hydroxyls, or carboxylic acids to the solid support structure, which can then form covalent bonds with the different amino acid residues present in the target protein structure (Datta et al., 2013; de Moraes et al., 2013, 2015; Homaei et al., 2013; Mohamad et al., 2015; Li Q. et al., 2017; Tao et al., 2019). Other procedures are based on non-covalent immobilization, like protein adsorption onto the solid support (Ma et al., 2015; Zhan et al., 2016), entrapment (Anguizola et al., 2016; Yang Y.-X. et al., 2017) and biospecific adsorption (Temporini et al., 2013), but they are more susceptible to protein desorption, particularly in on-line systems.

Immobilization can be carried out “*in situ*” (Chen X. et al., 2017; Tao et al., 2018a); that is, all the protein immobilization steps are conducted within the LC-suitable devices (columns, microcolumns, capillaries, disks, etc) or “in batch” (Habicht et al., 2015; Zhan et al., 2016), when the target protein is immobilized on the solid support for later packing. In the former case, the target protein structure can change (including enzyme inactivation) during the column packing process. However, the use of the “in batch” methodology to prepare bioaffinitity columns has spread considerably (see Table 1 for data about immobilization procedures that are used to prepare solid supports for frontal bioaffinity chromatography (FAC) assays).

**Table 1:** Literature studies that have used FAC assays to probe ligand-target interactions.

**Target**	**Solid support**	**Immobilization method**	**Ligands**	**References**
Human serum albumin (normal and glycated)	Nucleosil Si-300 silica	Covalent via Schiff base formation (*in situ*)	Chlorpropamide Glimepiride (FAC), warfarin, L-tryptophan, tamoxifen, and digitoxin in zonal elution	Matsuda et al., 2015; Tao et al., 2018a
Beta2-adrenoceptor	Silica gel	Covalent (In batch)	Salbutamol and terbutaline for characterization; bioactive compound in Shaoyao-Gancao decoction	Li Z. et al., 2017
Thrombin	IAM	Entrapment (*in situ*)	Ferulic acid, gallic acid, protocatechuiic acid, chlorogenic acid, and sinapic acid	Yang Y.-X. et al., 2017
Angiogenesis inhibitor Kringle 5	Silica gel	Oriented immobilization by histidine-tagged attachment	L-lysine, epsilon-aminocaproic acid (EACA), 7-aminoheptanoic acid (7-AHA), trans-4-(aminomethyl)cyclohexane carboxylic acid (AMCHA), and benzylamine	Bian et al., 2015
Cell membrane from rat brains containing dopamine receptor	Silica gel	Adsorption onto the solid support (In batch)	Dopamine, olanzapine, quetiapine, bupropion, and domperiodone	Ma et al., 2015
Human Purine Nucleoside Phosphorykase (HsPNP)	Capillary (open tubular and monolithic)	Covalent (*in situ*)	HsPNP inhibitors with different inhibitory potencies	de Moraes et al., 2014a
Membranes from cells containing A2A adenosine receptor subtype	IAM and open tubular capillary	Covalent (*in situ*) in the open tubular capillary and biospecific adsorption/entrapment in IAM support (In batch)	8-substituted-9-ethyladenines (four derivatives)	Temporini et al., 2013
Voltage-dependent anion channel isoform 1 (VDAAC-1)	Macroporous silica gel	Covalent immobilization on silica gel surface using phospholipid monolayer (In batch)	ATP, NADH, and NADPH	Li Q. et al., 2017
Cell membranes containing human α3β4α5 and α3β4 nicotinic receptors	IAM	Entrapment (In batch)	Epibatidine, nicotine, cytisine, nornicotine, and anabasine	Ciesla et al., 2016
Translocator proteins in mitochondrial transmembrane proteins from monkey skeletal muscle and human platelets	IAM	Adsorption/entrapment (In batch)	Dipyridamole and translocator protein ligands (PK11195, photoporphyrin IX, and rotenone)	Singh N. S. et al., 2017
Translocator proteins in mitochondrial transmembrane proteins from U87MG and HEK-293 cells	IAM	Adsorption/entrapment (In batch)	Dipyridamole, PK-11195, mesoporphyrin IX, photoporphyrin IX, and rotenone (translocator protein ligands)	Habicht et al., 2015
β2-adrenoreceptor	Silica gel	Covalent (*in situ*)	Protopine	Liu G. et al., 2017
Cell membranes containing α_1A_ adrenoreceptor from HEK293 cell line	Silica		Tamsulosin hydrochloride and seven alkaloids	Wei et al., 2017
Human serum albumin (normal and glycated)	Nucleosil Si-300 silica	Covalent via Schiff base formation (*in situ*)	Tolazamide	Tao et al., 2019
High epidermal growth factor from HEW293 cells	Silica	Adsorption (In batch)	Taspine derivatives (TPD7 and HMQ1611) and afatinib	Zhan et al., 2016
TEM-1 beta-lactamase	Silica gel	Covalent (*in situ*)	Beta-lactam antibiotics (cafelexin, penicillin G, and cefoxitin)	Chen X. et al., 2017

Zonal (linear and non-linear) chromatography and frontal affinity chromatography are the elution modes that are most often employed in bioaffinity chromatography assays. This section will discuss the application of zonal and frontal bioaffinity chromatography in on-line systems to probe and to characterize ligand-target protein interactions.

### Frontal Bioaffinity Chromatography

Frontal analysis involves continuous analyte infusion into the chromatographic column, so it generally requires larger sample injection volumes. In this approach, the experiments are carried out under dynamic equilibrium conditions, and the ligands (or the analytes as potential ligands) are continuously infused through the column. As the bioaffinity column stationary phase becomes saturated, the concentration of ligands eluting from the column increases gradually until a plateau is reached. Ligands break through the column at distinct times according to their concentration and affinity for the stationary phase (ideally, for the immobilized protein target). Therefore, the ligands can be ranked, and the binding constants can be precisely determined (Calleri et al., 2009, 2010; de Moraes et al., 2016).

**Figure 2:**
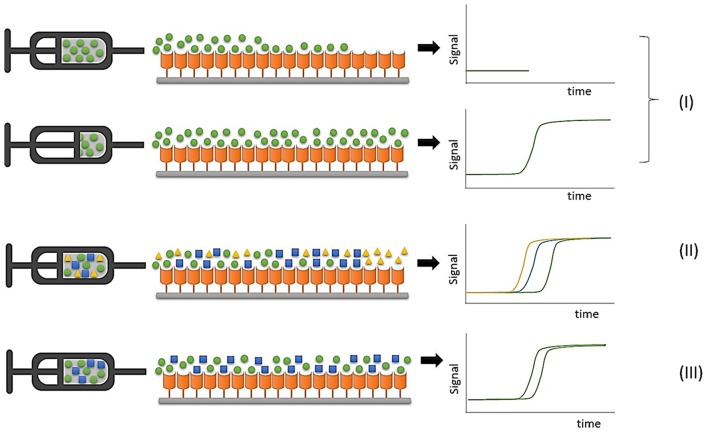
Representative illustrations of FAC experiments. (I) Direct assays to determine binding constants with an individual ligand infusion; (II) Ranking assays to classify compounds in mixtures; (III) Displacement experiments to evaluate ligand-protein interaction by monitoring a marker ligand.

FAC studies can be conducted by directly monitoring the ligand elution profile in an approach known as direct assay, in which the retention pattern is directly associated with ligand concentration and affinity for the immobilized protein target. Displacement studies are considered indirect assays because a known ligand is used as marker, and interaction between an analyte and the immobilized protein target is indirectly evidenced by a displacement of the marker elution profile due to competition by the binding sites, as illustrated in Figure 2.

In ranking experiments accomplished by FAC, during which mixtures of compounds are continuously infused into the chromatographic column containing the immobilized protein target, the detector should be able to discriminate the elution profile of each component of the initial mixture, and the use of a selective detector like electrospray ionization mass spectrometry is mandatory due to its ability to distinguish between different co-eluting m/z values (Slon-Usakiewicz et al., 2005; Ng et al., 2007; Calleri et al., 2011).

FAC methods allow ligand-target interactions to be determined in terms of dissociation (K_d_) or association (K_a_) constants, where K_d_ = 1/K_a_, through the basic FAC equation:

(1) $$(V-V_{0})=B_{t}\times \frac{1}{\left[L\right]+\;K_{d}}$$

Where B_t_ is the number of available binding sites, V is the ligand breakthrough volume, V_0_ is the breakthrough volume in the absence of the binding event, [L] is the ligand concentration, and K_d_ is the dissociation constant.

A frontal chromatography assay called modified staircase method is an alternative strategy to assess K_d_ and B_t_. The washing and equilibrium steps between the individual analysis of each evaluated concentration in FAC assays is time-consuming, and the modified staircase (or stepwise frontal analysis) (He et al., 2018) constitutes a promising method to determine the binding constants (de Moraes et al., 2016). In this assay, the ligand is sequentially infused until saturation by a series of low-to-high concentrations is achieved, forming a staircase pattern; with simultaneously infusion of a void marker at a fixed concentration. Columns containing human serum albumin (HSA) and alpha-1-acid glycoprotein (AGP) have been employed to determine the equilibrium dissociation constant K_d_ for warfarin- and digitoxin-HSA and verapamil- and tamsulosin-AGP interactions by direct FAC and stepwise frontal analysis. K_d_ values obtained through the different approaches correlate well with literature values, evidencing that the modified staircase method can be applied to assess the binding constants (He et al., 2018).

The interaction between three β-lactam antibiotics (penicillin G, penicillin V, and cefalexin) and bovine serum albumin (BSA) has been investigated by FAC–UV (Li et al., 2016), by delivering 200 mg of the target protein through a stainless-steel column (50 × 4.6 mm I.D.) to immobilize the target covalently. When different ligand concentrations are infused during the FAC experiments, the binding constants for the ligand-target protein interaction (K_a_) and the number of binding sites in the stationary phase can be determined. Displacement experiments were conducted to investigate the binding site of the selected ligands: K_a_ values of all the three β-lactam antibiotics decrease in the presence of warfarin, an anticoagulant that binds to binding site 1 in subdomain II A, demonstrating that the binding sites of these drugs to BSA are mainly located therein.

FAC–MS has been employed to assess the adsorption data of three drugs (salbutamol, terbutaline, and pseudoephedrine) and the beta-2-adrenoreceptor (β_2_-AR) attached to polystyrene amino microspheres (Li et al., 2018). Adsorption data of the selected drugs obtained by FAC–MS and site-specific studies have helped to investigate the adsorption models for the binding of each ligand through adsorption energy distribution calculations. In addition, FAC–MS competitive assays have been described as an efficient strategy to screen β_2_-AR ligands by displacement experiments.

BSA has also been covalently immobilized on penetrable silica microsphere through an “in batch” methodology (Ma et al., 2016), and the ability of the BSA-containing stationary phase to separate D- and L-tryptophan was assessed in the zonal elution mode, to evidence that the bioaffinity column is enantioselective. Further FAC–UV studies helped to probe the interaction between imatinib mesylate and BSA, allowing the number of active binding sites on the stationary phase and K_a_ to be determined.

Alpha_1_-acid glycoprotein (AGP) has been immobilized “*in situ*” by physical entrapment in microcolumns packed with hydrazide-activated porous silica (1 cm × 2.1 mm) and applied in frontal and zonal elution studies to investigate the binding of different ligands. Frontal studies revealed the association equilibrium constant (K_a_) and the moles of binding sites for the AGP-carbamazepine interaction. FAC experiments with a control microcolumn (without entrapped AGP) pointed to some non-specific interactions with the support, which has frequently been observed in microparticulate supports (Xuan et al., 2010; Anguizola et al., 2016). Microcolumn heterogeneity can stem from the microparticulate support being incompletely coated with the target protein, to result in specific binding regions (ligand-target protein) and non-specific interaction regions (ligand-solid support) (Muller and Carr, 1984; Tweed et al., 1997).

FAC provides a lot of information about the immobilized target protein and the ligand-target protein interaction recognition. As can be noted in Table 1, FAC is a versatile approach that enables various proteins and ligands to be studied by different methods. Some studies have explored all the possibilities of this elution approach to probe ligand-target protein interactions through different assays (ligand characterization by direct assays, ligand ranking, displacement) (Temporini et al., 2013; de Moraes et al., 2014a; Ciesla et al., 2016; Chen X. et al., 2017; Yang Y.-X. et al., 2017), while other studies have used this elution mode to characterize the physicochemical properties of the target protein-containing solid support associated with the zonal elution mode for affinity and displacement studies (Habicht et al., 2015; Ma et al., 2015; Guo et al., 2017; Liu G. et al., 2017; Tao et al., 2018a). The next section discusses the potential of the state-of-the-art zonal elution to probe ligand-target protein interactions.

### Zonal Chromatography

In liquid chromatography, zonal elution encompasses injecting a small amount of analyte through a column and using an online detector to monitor the analyte elution time or volume. This elution mode has great potential to probe ligand-target protein interactions when immobilized target protein-containing stationary phases are employed. Compared to the amount of target protein that is immobilized on the solid support, the amount of injected ligand is negligible (a requisite for linear elution), however, non-linear elution conditions have been also employed to probe ligand-target protein interaction by zonal chromatography (Vanzolini et al., 2013b; Li Q. et al., 2015, 2017; Liang et al., 2018).

Although zonal elution involving on-line detectors has been considered the most common approach, literature papers have also described some off-line assays in which a fraction from the zonal elution experiment is collected and analyzed on an off-line detector, mainly when the experiment is conducted in a low-performance target-containing column (Tao et al., 2018b).

Affinity assays by zonal chromatography can provide information on the ligand-target protein interaction by direct measurements or competition experiments. Direct measurements entail peak retention time monitoring, retention factor determination, or peak profile evaluation. As for competition experiments, a known ligand is added to the mobile phase, and an analyte (second ligand or potential ligand) is injected into the chromatographic system, to monitor the time or volume that is necessary to elute the analyte from the bioaffinity column (Zheng et al., 2014; Tao et al., 2018b). Competition assays are also a useful tool to investigate the binding site of different ligands.

The binding event can be evaluated by injecting a small amount of the ligand into the bioaffinity column and monitoring the elution time (to determine the peak retention time and retention factors) and/or peak profile. When the ligand-target protein interaction occurs through fast association and dissociation kinetics, the ligand retention time should be directly associated with the ligand-target protein interaction strength and the amount of immobilized target protein (Gargano et al., 2014; Zhan et al., 2016; Ohlson et al., 2017). The elution time (or volume) should be monitored along with a void volume marker and, to obtain reliable results, the ligand-target protein interaction specificity should be investigated by using a control column (solid support without the immobilized target protein or with the immobilized inactive target protein). When the elution times of different ligands are compared, they can be ranked according to their affinity for the immobilized target protein. Equations to explore the ligand-target protein binding event are available in recent reports (Zheng et al., 2014; Tao et al., 2018b).

Competition assays are performed by employing a displacer agent in the mobile phase, which shifts the ligand retention as both compounds (the ligand and the displacer agent) compete for the same binding site on the immobilized target protein surface (Gao et al., 2014; Matsuda et al., 2015; Anguizola et al., 2016; Liu G. et al., 2017; Wei et al., 2017; Tao et al., 2018a, 2019).

Information regarding the binding site for the ligand-target protein interaction and the nature of this interaction can be assessed by meticulously examining the experimental conditions in zonal experiments: mobile phase pH, polarity, and ionic force, presence of other ligands (displacing or competing agents), temperature, ligand type, and target protein (Zheng et al., 2014).

Ligand binding to the target protein can be monitored and characterized by the peak profile obtained in the zonal elution assays. In this approach, a small ligand sample is injected into the bioaffinity column and the control column. The eluted peak width is used to gather information regarding the ligand-target protein binding kinetics. This methodology, also known as band-broadening measurement, encompasses the plate height method and the peak profile method (Chen et al., 2009; Yoo and Hage, 2011; Hage, 2017). Other strategies like the peak decay, peak fitting, and split-peak methods can be employed to investigate the ligand-target protein binding event kinetics (Bi et al., 2015; Beeram et al., 2017; Anguizola et al., 2018; Liang et al., 2018), but they are outside the scope of this review.

Zonal elution has also been applied to monitor on-line enzyme activity in ligand screening assays, resulting in reliable and specific assays that allow the biocatalysis product to be directly quantified (da Silva et al., 2012; de Moraes et al., 2013; Calil et al., 2016; Lima et al., 2016; Magalhães et al., 2016; Ferreira Lopes Vilela and Cardoso, 2017; Cornelio et al., 2018; Vilela et al., 2018; Seidl et al., 2019). This approach prevents interferences in inhibitors screening and furnishes reliable data concerning the substrate- and inhibitor-enzyme binding. Recently, the activity of two classes of acetylcholinesterases (AChE) from *Atta sexdens* immobilized on capillary columns was monitored by directly quantifying choline, obtained from the hydrolysis of acetylcholine, which is the AChE natural substrate. The traditional colorimetric assay (Elman method), which employs acetylthiocoline as substrate, results in inverse AChE-substrate affinities for the two different classes of AChE (Dos Santos et al., 2019). This evidences that direct assays are important to monitor the enzyme activity and to characterize binding affinities.

Some recent papers on zonal bioaffinity chromatography for enzyme activity assays have used different protein targets simultaneously in the chromatography system to yield selectivity and specificity results fast. A simultaneous on-flow enzyme assay that uses two different immobilized enzymes (AChE and butyrylcholinesterase) in parallel in the chromatography system has been recently reported. In this approach, the inhibitory activity of an analyte can be simultaneously evaluated for both enzymes by using two 10-port/two-position switching valves with a single injection in a process that takes <6 min (Seidl et al., 2019).

On-line bioaffinity chromatography studies have been employed to isolate ligands from mixtures by means of different strategies. In 2014, Forsberg and Brennan used covalently linked adenosine deaminase (ADA) columns to isolate and to extract inhibitors from complex mixtures by combining activity- and affinity-based assays (Forsberg and Brennan, 2014). In a first moment, this strategy involved screening different mixtures in an activity-based assay. After that, the identified bioactive mixtures were infused in an ADA-containing monolithic silica capillary column until MS detector saturation was achieved, which is followed by a wash step to remove unbound compounds. The retained ligands were eluted with a harsh wash and identified by MS/MS.

More recently, multidimensional liquid chromatography systems (2D-LC) have been explored to isolate and to extract ligands from complex matrixes through fully automated systems (Han et al., 2013; Jia et al., 2016; Wu et al., 2016; Guo et al., 2017; Wang et al., 2017; Wang X.-Y. et al., 2018). In this context, an immobilized xanthine oxidase microcolumn was used to selectively retain bioactive compounds from *L. macranthoides* extract and to transfer them to an analytical column, where the identified inhibitors are isolated. The 2D LC–MS/MS system enabled nine bioactive compounds from *L. macranthoides* to be rapidly isolated (Peng et al., 2016).

Comprehensive two-dimensional chromatography was applied to investigate bioactive compounds from *Indigo naturalis*, a famous Traditional Chinese Medicine that is used in the treatment of leukemia in China. Columns containing active components and membrane receptors from the K562 cell line were used in the first dimension to retain the bioactive compounds selectively and to transfer them to the second dimension via two trap columns, with an C_18_ analytical column with detection by QqTOF. Three active compounds were characterized, and their anti-leukemia effect was confirmed by cell viability and cell apoptosis assays (Wu et al., 2016).

A 2D-LC–MS screening platform was designed to isolate AChE ligands from *Corydalis yanjusuo* extracts selectively. To this end, monolithic AChE capillaries were used as the bioaffinity columns in the first dimension. To avoid false results caused by non-specific binding, control experiments were run simultaneously with a denatured enzyme column. Eight AChE ligands were isolated from this experiments and their inhibitory activities were confirmed by activity-based assays (Wang L. et al., 2018).

Columns with solid supports containing cell membranes from rat hearts (normal and pathological tissue) have been employed in an on-line chromatography system (comprehensive 2D using a 10-port-dual-position valve) to screen specific therapeutic agents from *Acontium carmichaeli* that can counteract doxorubicin-induced heart failure (Chen et al., 2014).

Advantages of the zonal elution mode for bioaffinity chromatography include versatility in terms of methodology (as seen from the numerous approaches presented and discussed herein), use of a small amount of sample, and possibility of full automation systems even during control experiments conducted in parallel with the binding assays.

## Off-Line Static Approaches

Considered as one of the most efficient and convenient methods to separate potential ligands from complex mixtures, the affinity-based screening assay can be applied to investigate multiple interacting pairs involved in biological systems such as antigen-antibody, receptor-ligand, enzyme-inhibitor/activator, and protein-protein (Hage et al., 2012). These assays employ many macromolecular targets, like receptor, enzyme, transport protein, and cell membrane (Zhuo et al., 2016).

**Figure 3:**
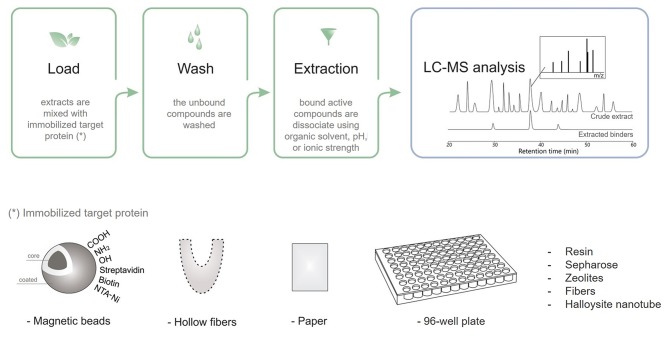
Schematic ligand fishing approach applied to screen active compounds from complex mixtures.

Taking advantage of diverse targeting immobilization methods and several analytical approaches, ligand fishing strategies have emerged as practical and effective procedures to fish out ligands from complex mixtures. Ligand fishing experiments essentially rely on the fact that any compound with affinity for the immobilized target protein is retained (affinity selection) for further analysis, while non-binding compounds remain in the extract/supernatant and can be discarded (Zhuo et al., 2016). Briefly, this approach is carried out by an immobilization procedure, followed by an incubation step, washing to separate binders from non-binders, and binder desorption and characterization (Figure 3). Analytical approaches based on LC–MS are generally employed to obtain a chemical profile of the ligands with affinity for the immobilized target protein. These ligands can be structurally characterized either directly, by conducting LC–HRMS of the ligand-containing fraction, or through targeted LC–PDA–HRMS–SPE–NMR analysis of the crude extract (Arai et al., 2009; Wubshet et al., 2015; Cieśla and Moaddel, 2016). The ligand-bound complex is usually separated from the unbound compounds by approaches like ultrafiltration, dialysis, affinity purification, size-exclusion chromatography, magnetic separation, and hollow fiber adsorption, among others (Moaddel et al., 2007; Song et al., 2015; Wang et al., 2015; Zhuo et al., 2016; Hu et al., 2018). The applied method will depend on the support material.

The use of ligand fishing assays has increased in the search for bioactive natural products. Target proteins have been immobilized on various supports, including magnetic beads (de Almeida et al., 2017; Wang Z. et al., 2018; Tang et al., 2019), quantum dots (Hu et al., 2018), hollow fibers (Chen L. et al., 2017), nanotubes (Wang et al., 2015), and monolithic silica (Forsberg and Brennan, 2014).

### Magnetic Supports

Magnetic supports or magnetic particles (MPs) are also known as magnetic beads (MBs), micro- and nanosized magnetic beads, paramagnetic beads (PBs), ferrofluids, and magnetic fluids, and they are an excellent support option (Marszałł, 2011). Protein immobilization on MBs offers the following advantages: stable immobilization (protein-protein complexes remain intact on the protein-coated MB surface) and easy magnetic isolation with the use of external magnets (which prevents contact with the analyte solution) (Marszałł et al., 2008; Zhuo et al., 2016). Ligand fishing assays based on magnetic particles are an outstanding tool to identify bioactive constituents in plant extracts (Zhuo et al., 2016; Tang et al., 2017, 2019; Yang X.-X. et al., 2017; Vanzolini et al., 2018b; Wang Z. et al., 2018; Wubshet et al., 2019; Zhang et al., 2019).

One advantage of using MBs is that they allow low-affinity ligands and secondary metabolites of low abundance, but with great affinity for the target protein to be identified. Such ligands and metabolites are normally overlooked when other screening techniques are employed.

Moaddel et al. were the first to apply MBs for ligand fishing (Moaddel et al., 2007). By using human serum albumin (HSA), they showed that a protein-coated MB fishes out known binders from a mixture of binders and non-binders. HAS-coated MBs have affinity selection, as demonstrated in the study involving control beads (blank) without immobilized HSA. The results reported by these authors correlate with data previously reported for bioaffinity chromatography assays. A rate limiting step of ligand fishing experiments is the amount of protein that is required for the successful fishing experiments, typically 50 μg (Cieśla and Moaddel, 2016). Since the publication of this pioneering work, several proteins have been immobilized on MBs and used to disclose ligands from complex mixtures (Cieśla and Moaddel, 2016; Zhuo et al., 2016; Liu et al., 2017a; Yang X.-X. et al., 2017).

de Almeida et al. (2017) extracted angiotensin-converting enzyme (ACE) from bovine lung, purified it, and covalently immobilized it on modified ferrite magnetic beads (ACE-MBs) to fish out not only the reference inhibitor, but also one peptide from a pool of tryptic digested BSA.

A fluorimetric ACE inhibitor assay was developed by immobilizing ACE on anti-FLAG antibody-coated MBs by using a 96-well microplate operation, fluorescence detection, and a two-step screening assay (Tang et al., 2019). On the basis of primary screening, five compounds exhibited inhibition rate >25% [(+)-tetrandrine, fangchinoline, narcissoside, epiberberine, and verbascoside]. Because natural products can affect the fluorescence, the product standard solution was mixed with test compounds and then derivatized for fluorescence detection to evaluate the fluorescence change in the second step. A fluorescence alteration over 25% was considered as interference. Fluorescence intensification can lead to false-negative results, while reduced fluorescence can provide false-positive results. According to the findings, ten compounds, including the five hits from the preliminary screening, decreased the fluorescence by over 25%, but no compound intensified the fluorescence. Epiberberine and fangchinoline displayed better ACE inhibition activity with IC_50_ values of 52.61 ± 4.12 and 97.48 ± 5.34 μM, respectively.

Membrane-bound α-glucosidase enzyme-coated MBs have successfully fished out four natural α-glucosidase ligands from *E. catharinae* (Wubshet et al., 2015). Designed chitosan-enriched magnetic composites were also used to immobilize α-glucosidase and to disclose enzyme inhibitors from extracts of Traditional Chinese medicines (TCMs) and vegetables (Liu et al., 2017b).

AChE has been successfully immobilized on MBs to screen compounds from plant extracts (Vanzolini et al., 2013b, 2018b). Moreover, *Electrophorus electricus* (*eel*) AChE-coated amino-modified paramagnetic beads have been applied in an affinity-based ligand-fishing assay to discover bioactive peptides from complex protein mixtures from black mamba venoms. Tryptic digestion followed by nano-LC-MS analysis of the material recovered from black mamba venom identified the peptide with the highest AChE-binding affinity as dendrotoxin-I, a pre-synaptic neurotoxin that had not been known to interact with AChE (Vanzolini et al., 2018a).

α-Amylase-coated magnetic nanoparticles have been employed to fish out ligands from *Garcinia xanthochymus* extracts, which led to three biflavonoids being identified as inhibitors (Li et al., 2014).

Deng et al. (2014) established a screening assay based on magnetic Fe_3_O_4_@SiO_2_-COX-2 ligand fishing combination with LC–DAD–MS^n^ to screen and to identify COX-2 inhibitors from green tea. The authors fished out eight catechins with COX-2 binding activity, two of which for the first time. For Fe_3_O_4_@SiO_2_-COX-1, four curcuminoids were isolated as main COX-1 inhibitors (Zhang et al., 2014).

Zhang et al. (2019) used monoamine oxidase-A (MAO-A) immobilized on the MB surface (MAO-A-MBs) to conduct ligand fishing and LC-HRMS to characterize ligands. Seven compounds (tetrahydrocolumbamine, protopine, jatrorrhizine, glaucine, tetrahydropalmatine, palmatine, and dehydrocorydaline) with high binding affinity for MAO-A were identified from the *Corydalis Rhizome* extract ethyl acetate fraction. The immobilized MAO-A activity remained over 80% after storage at 4°C for about seven days.

Glycogen synthase kinase-3β (GSK-3β) was immobilized on MBs and used to screen the inhibitory activities of 15 TCM extracts. Three of these TCMs, *Euonymus fortunei, Amygdalus communis*, and *Garcinia xanthochymus*, exhibited high inhibitory activity (inhibition rate > 90%). A new GSK-3β inhibitor, called fukugetin, with an IC_50_ value of 3.18 ± 0.07 μM was discovered in the *G. xanthochymus* extract. The immobilized enzyme was reused 10 times and remained stable at 4°C for 4 days (Li Y. et al., 2015).

de Moraes et al. (2015) immobilized cellular prion protein (PrP) on MB surface and applied it to isolate ligands in a mixture of compounds by employing LC-MS. The anti-prion compound quinacrine, an inhibitor of PrP aggregation, was isolated.

Recent developments in biological systems and overall clinical experience have suggested that, due to homeostatic nature, single-target drugs may not always induce the desired effect on the entire biological system even if they successfully inhibit or activate a specific target (Pang et al., 2012). Thus, the concept and the strategy of developing multi-target or multi-component drugs have recently been proposed (Zimmermann et al., 2007; Wang et al., 2012). Although countless valuable studies have reported the use of approaches based on protein-coated MBs that can identify active compounds from medicinal plant extract fast, most previous research has focused on ligand binding to a single target.

Tao et al. (2013a) developed a multi-target strategy to screen bioactive compounds from a botanical drug by immobilizing multiple targets (maltase, invertase, and lipase) on the MBs through covalent linkage. This approach was applied to screen ligands from the Chinese medicine “Tang-Zhi-Qing,” which is used to treat type II diabetes in China. To this end, the authors placed MBs immobilized with different targets (e.g., maltase, invertase, and lipase) into three connecting chambers separately and pumped the unpurified botanical drug into the chambers by means of a peristaltic pump. They found that incubation leads the ligands to bind to the targets, as attested by the LC-MS analysis conducted after the wash and extraction steps. Therefore, this approach successfully fished out seven ligands which bound to the three immobilized target enzymes. Even though paeoniflorin and salvianolic acid B could bind to the enzymes, they showed no maltase, invertase, or lipase inhibitory activity at all. Compared to classic screening methods, the proposed approach can rapidly identify bioactive compounds that specifically bind to different targets, which could enhance the discovery of active compounds.

Imaduwage et al. (2016, 2017) described a detection strategy to fish out strong binders only. In this approach, the inhibitors/binders were incubated with the protein (binding experiment) and, separately, with blocked beads (control experiment). After incubation time the non-binding compounds from both experiments were removed and analyzed by LC-MS. Strong binders were identified by comparing the spectral data of the control and binding experiments. Table 2 list other applications of MB-bioreactors.

**Table 2:** Ligand fishing strategies based on MB-bioreactors on TCM.

**Target**	**Plant**	**Method**	**Active compounds**	**References**
α-amylase	Garcinia xanthochymus	Enzyme coated magnetic nanoparticles	GB2a glucoside, GB2a, and fukugetin	Li et al., 2014
Xanthine oxidase	*Radix Salviae Miltiorrhizae*	Affinity selection-based 2D chromatography coupled with LC-MS	Salvianolic acid C and Salvianolic acid A	Fu et al., 2014
Cyclooxygenase-1 (COX-1)	*Turmeric*	Enzyme coated magnetic nanoparticles	Curcumin, demethoxycurcumin, bisdemethoxycurcumin, and 1-(4-hydroxy-3,5-dimethoxyphenyl)-7-(4-hydroxy-3-methoxyphenyl)-(1*E*,6*E*)-1,6-heptadiene-3,5-dione	Zhang et al., 2014
Cyclooxygenase-2 (COX-2)	*Green tea*	Enzyme coated magnetic nanoparticles	(*-*)-Epigallocatechin-3-(3″-*O*-methyl)-gallate and (*-*)-epicatechin-3-(3″-*O*-methyl)-gallate	Deng et al., 2014
GSK-3β	*Euonymus fortunei* and *G. xanthochymus*	Magnetic beads	Fukugetin	Li Y. et al., 2015
α-Glucosidase s	*Eugenia catharinae*	Enzyme coated magnetic bead	5-(2-Oxopentyl)resorcinol 4-*O*-β-D-glucopyranoside, 5-propylresorcinol 4-*O*-β-D-glucopyranoside, 5-pentylresorcinol 4-*O*-[α-D-apiofuranosyl-(1 → 6)]-β-D-glucopyranoside, 5-pentylresorcinol 4-*O*-β-D-glucopyranoside, 4-hydroxy-3-*O*-methyl-5-pentylresorcinol 1-*O*-β-D-glucopyranoside, and 3-*O*-methyl-5 pentylresorcinol 1-*O*-[β-D-glucopyranosyl-(1 → 6)]-β-D-glucopyranoside	Wubshet et al., 2015
Neuronal cells	*Radix Polygalae*	PC12 cell membrane chromatography-LC–(Q)TOF-MS	Onjisaponin B	Wu et al., 2015
Lipase	*Magnoliae cortex*	Lipase-adsorbed nanotube combined with LC–MS analysis	Magnotriol A and magnaldehyde B	Wang et al., 2015
SIRT6	*Trigonella foenum-graecum*	SIRT6-coated magnetic beads	Orientin and 17 other compounds	Singh et al., 2014
α-glucosidase	*Codonopsis pilosula, Rhizoma coptidis, Forsythia, Radix hedysari, Semen allii tuberosi, Radix isatidis, Rhizoma atractylodis macrocephalae, Glycyrrhiza uralensis Fisch, Folium Mori, Radix Ophiopogonis, Radix Puerariae, Platycodon grandiflorus, Radix Astragali, Radix Notoginseng, Radix et Rhizoma Rhei, Cortex Moutan, Phellodendron amurense, Eucommia ulmoides*	α-glucosidase- coated Fe_3_O_4_/CS/GA/α-Glu nanoparticles coupled to capillary electrophoresis		Liu et al., 2017a
Neuraminidase		surface of magnetic beads	luteolin-7-O-β-D-glucoside, luteolin, 3,5-di-O-caffeoylquinic acid, and 3,4-di-O-caffeoylquinic acid	Zhao et al., 2018

### Other Supports

Over the last decade, many nanomaterials, such as carbon and TiO_2_ nanotubes, have been used as microextraction medium for selective enrichment with specific compounds (Zhuo et al., 2016).

Hollow fibers have been widely used to pretreat samples (Yang et al., 2015). During hollow fiber adsorption, screening targets are immobilized on the inner wall of a hollow fiber via physical adsorption (Yang X.-X. et al., 2017). Physical adsorption onto hollow fibers is easier and prevents protein structural modification during the chemical binding process (Zhuo et al., 2016).

Wang et al. (2015) were the first to establish lipase-adsorbed halloysite nanotubes (HNTs) for ligand fishing from natural products extracts. They reported that three flavonoids were rapidly isolated and identified as lipase ligands from *Lotus leaf* extract (Tao et al., 2013b). Later, the same research group successfully fished out four neolignan compounds from *Magnoliae cortex* extracts. The target protein adsorbed onto hollow fibers had short activity time, and only a few targets could be adsorbed, so the sensitivity of this method was limited.

Hollow fiber adsorption has been used to screen ligands of living cells, cell membranes, organelles, and enzymes (Liu et al., 2014; Chen et al., 2016; Zhang Q. et al., 2018). Chen L. et al. (2017) developed a multi-target screening strategy to identify bioactive components in TCMs by using hollow fiber-based ligand fishing (HFLF) followed by identification of the ligands dissociated from the target-ligand complexes by LC–MS. After individual microporous U-bent hollow fibers containing the enzymes α-glucosidase and ACE were prepared, the hollow fibers filled with the enzymes were heat-sealed, and their open end was immersed into the Ganjiang Huangqin Huanglian Renshen Decoction (GHHRD) extract, which include *Rhizoma zingiberis, Rhizoma coptidis, Radix Scutellariae*, and *Radix Ginseng* extracts. This study identified coptisine as the α-glucosidase ligand and baicalin as the ACE ligand. Berberine was found to be a dual inhibitor of α-glucosidase and ACE.

Xu et al. (2019) applied cellulose filter paper (CFP) as carrier of cell membrane (CM) and developed a novel CM-coated CFP. They used this approach to fish active compounds from *Angelica dahurica* extracts. Three potentially active compounds, including bergapten, pabulenol, and imperatorin, were fished out and identified.

Lau et al. (2015) reported the use of resin to immobilize different enzymes (p97, also known as valosin containing protein (VCP) or cdc48), His6-p97, His6-HSC70, HSPA1A13, and malate dehydrogenase (MDH) by affinity method. The authors used these resin-supported enzymes to isolate small molecules from natural products. LC was used to analyze each elution fraction, and unique peaks were subjected to HRMS. As control, the authors used a resin without protein (data not shown) or recombinant *E. coli* FtsZ (which served as a non-specific protein control).

Kang et al. (2014) described the use of a resin-supported target protein p97 to isolate three natural products (rheoemodin, hydroxydehydroherbarin and phomapyrrolidone A) from crude extracts of the fungal strain *Chaetomium globosum*, endolichenic fungus, *Corynespora* sp., and the endophytic fungal strain *Phoma* sp., respectively, each with a different mechanism of action (Kang et al., 2014). Although the authors carried out these studies by a static approach, they called them as “functional chromatography.”

Tao et al. (2016) used zeolites and MBs to immobilize AChE. The resulting AChE-zeolites and AChE-MBs were used to extract ligands from *Corydalis yanhusuo* crude extract by LC-HRMS. While zeolite-AChE fished out 14 inhibitors, AChE-MBs helped to isolate 10 inhibitors. A comparison between zeolite and MBs approaches discloses different immobilization methods; that is, adsorption and covalent binding for zeolite and magnetic nanoparticles, respectively. The AChE-zeolite immobilization ratio was three times larger than the AChE-MB immobilization ratio, which means that more AChE could be immobilized on the same amount of zeolite. Although both AChE-zeolite and AChE-MBs could be reused, AChE can be recycled from the zeolites by desorption. As AChE-zeolites only require AChE and zeolite, they are considered environmentally friendly and inexpensive.

The S-transferase-tagged human PPARγLBD (GST-hPPARγLBD) was bacterially produced and directly applied to a 96-well filter plate pre-packed with glutathione sepharose. Due to the strong bioaffinity between GST and glutathione, the GST-hPPARγLBD could selectively attach to glutathione sepharose, to achieve oriented immobilization and rapid purification. The produced 96-affinity column array was used in an LC–HRMS to fish PPARγ ligands from the extracts of *Magnolia officinalis* (Zhu et al., 2017). PPARγ-functionalized affinity chromatography provides excellent selectivity and sensitivity for fishing.

Despite the aforementioned advantages of fishing assays, many challenges remain, including effective ligand desorption from the target and non-specific binding, which can interfere in the identification of real active compounds. Therefore, experimental testing with blank material is always necessary (Zhuo et al., 2016). During immobilization, the target protein can be denatured or have its three-dimensional configuration altered. This culminates in mild binding or even no binding with the active compounds. To maximize protein immobilization and to improve screening sensitivity, immobilization conditions should be optimized for every selected target (Yang X.-X. et al., 2017).

Concerning the drawbacks of the affinity-based method, it requires pure target protein and modulation of each fishing assay step (see Figure 3) for each new target and, most of the time, for each natural product library. This is because the extraction step also depends on the ligand interaction mode. Moreover, the greatest challenge is to characterize the structure of the ligands identified in natural terrestrial and marine extracts: only small amounts are obtained, the structure is complex, several spectra are generated, and databases lack more curated information.

## Conclusions

Zonal and frontal bioaffinity chromatography assays have been successfully employed to identify and to characterize ligands from synthetic combinatorial libraries. In spite of their higher selectivity, due to their lower chromatography efficiency their application for identifying ligands in terrestrial or marine natural product extracts have been hampered. Efforts have been directed toward overcoming, however, these drawbacks and a list of innovative approaches based on bioaffinity retention has been designed. The possibility of hyphenating chromatographic systems to a myriad of mass spectrometers has been well-explored. In these approaches, the complicating factor has been to characterize the structures of the ligands identified on-line. Moreover, it sometimes not easy to adequate the bioaffinity column mobile phase with the one needed for ionization.

To overcome these shortcomings, the use of off-line bioaffinity devices has been expanded in order to isolate ligands for further chemical structure characterization. LC–SPE–NMR has already demonstrated its utility in the structural characterization of isolated ligands by going back to the crude extract chromatograms.

In this context, LC–HRMS is well-suited to high throughput analysis. Nevertheless, the generated databases are not searchable with raw data, and the amount of produced data is too large for manual analysis. As in the case of genomics and proteomics, which use an integrated platform, consolidation of the recently created Global Natural Products Social Molecular Networking (GNPS; http://gnps.ucsd.edu) will surely increase productivity in one of the most difficult steps of the bioaffinity assays.

The ability to determine not only the affinity and the kinetics, but also the structure of a hit compound directly from a complex mixture has accelerated lead identification and encouraged the use of solid-supported protein platforms.

## Author Contributions

MM designed the review, wrote the section about the on-line assays, and reviewed the whole manuscript. CC discussed the structure of the manuscript and wrote the section about the off-line assays. QC discussed the structure of the manuscript, wrote the introduction and conclusion sections, and reviewed the whole manuscript.

### Conflict of Interest

The authors declare that the research was conducted in the absence of any commercial or financial relationships that could be construed as a potential conflict of interest.
